# The Inflation Surge of 2021–22: Scarcity of Goods and Commodities, Strong Labor Markets and Anchored Inflation Expectations

**DOI:** 10.1007/s10272-022-1036-7

**Published:** 2022-04-08

**Authors:** Ángel Ubide

**Affiliations:** Head of Economic Research for Global Fixed Income, Citadel LLC, 131 South Dearborn Street, Chicago, IL 60603 USA

**Keywords:** E31, E43, E52

## Abstract

The key to understanding the series of supply shocks that have hit inflation is the nature of the COVID-19 recession.

Global inflation has increased to levels not seen in 40 years. This increase has been widespread: In February 2022, year-on-year headline inflation was 7.9% in the US, 5.9% in the eurozone and 6.2% in the UK, and it is likely to peak in the 8%-9% range. Excluding food and energy, year-on-year inflation was 6.4% in the US, 2.7% in the eurozone and 5.2% in the UK.[1] Other measures of underlying inflation, such as median or trimmed inflation, have showed increases of a similar magnitude, suggesting that price increases are becoming widespread and no longer concentrated on a few items. However, while inflation is a global phenomenon, some countries have bucked the trend: Year-on-year headline inflation in Japan, for example, was just 0.9% in February 2022.

This acceleration in inflation, which started around mid-2021, has been a surprise, after a few decades of very low inflation. This paper looks at the drivers of the inflation surge, its prospects and its implications for monetary policy. The discussion is focused on the US, but the main conclusions also apply to the EU and the UK: While the upside risks to inflation are higher than they have been in decades, the appropriate policy mix inflation will likely stabilize around the target.

## Why has inflation increased? A series of unfortunate events

We use the Phillips curve as an organizing principle for this discussion: Inflation would be a function of slack, inflation expectations, and supply shocks. In this framework, supply shocks are, by definition, short term and transitory, while slack averages out over the business cycle. Therefore, over the medium term, the Phillips curve approach implies that inflation would converge towards inflation expectations, and changes in inflation expectations have permanent effects on inflation.[2] Two hypotheses, not mutually exclusive, may explain the recent surge in inflation.

One hypothesis argues that a series of supply shocks have caused the surge in inflation. This surge would therefore be transitory yet it could be very persistent, because with a series of successive shocks it could take an extended period of time until all the shocks fade away. The other hypothesis argues that the surge in inflation is permanent, driven by excessively expansionary demand policies that generated too rapid an erosion of slack and lifted inflation expectations.

The two hypotheses can be difficult to tell apart in real time if, as it is the case, a series of simultaneous but unrelated price shocks affect a wide range of sectors of the economy. The breath and scope of these price shocks make them observationally equivalent to a macro shock to inflation, driven by demand growth outstripping supply. But the implications of each hypothesis are very different. This article analyzes the three components of the Phillips curve as way to tell which of the hypothesis is more valid.

## Transitory supply shocks: Scarcity of goods and commodities

The key to understanding the series of supply shocks that have hit inflation is the nature of the COVID-19 recession. It was not a standard recession triggered by overheating or by financial instability. Instead, it was triggered by a health shock that forced policymakers to shut down the economy, and suddenly put it in an induced economic coma. To support the economy during the coma, large fiscal and monetary policy easing packages were deployed with the objective of supporting incomes for those out of work, and minimizing hysteresis among workers and firms

1 These figures are not exactly comparable as the composition of the index differs across regions. “Inflation” refers to CPI in the US and to HICP in the eurozone and the UK.

* The author would like to thank Rania Papageorgiou, Giuseppe Vera and the macro group at the Peterson Institute for International Economics for comments and suggestions. The views or opinions expressed in this contribution are solely those of the author and do not reflect the views, policies or positions of the author’s employer, Citadel.

**Ángel Ubide**, Citadel LLC, Chicago, IL, USA.

2 This characterization fits with the empirical evidence that suggests that, to forecast inflation over a 12-month horizon, the critical inputs are the next release and the medium-term anchor (see Faust and Wright, 2019).

so that the disruption to the structure of the economy was minimal.

This policy strategy was very successful. As a result, the economic reopening was also very abrupt, and the economy bounced back very fast. But while economic policies succeeded at preventing a deterioration of potential output, the composition of output changed in an unexpected way: The risk of COVID-19 contagion penalized the consumption of services, triggering a large shift in the consumption of goods vs services.[3] In graphic terms, people suddenly stopped going to the gym and bought a bicycle. This meant that the global supply of durable goods became utterly insufficient overnight. Something similar happened to the production of semiconductors, the demand for which skyrocketed well above available supply due to, among other reasons, the need to manufacture all the computers needed to work from home. Since semiconductors are a critical input for the car production, this generated a global scarcity of cars.[4] Moreover, the global trading system and supply chains, optimized for efficiency and with little slack, broke down in multiple places with a global shortage of containers and trucks, and were not able to handle this sudden surge in global goods demand (see the discussion in Celasun et al., 2022). The result was a global supply bottleneck which, combined with robust demand growth, generated severe scarcity effects in goods markets, affecting multiple items in the CPI basket.

Scarcity effects generate non-linear increases in prices: the same amount of demand generates an outsized increase in prices. For example, with car inventories at all-time lows, the price of used cars in the US increased by almost 50% in 2021. Figure 1 illustrates the outsized impact of the price increases in cars on US inflation. While cars represent less than 10% of the CPI basket, it has generated about 50% of core CPI inflation. The counterfactual is clear: Had demand been better distributed across goods and services, the same amount of demand would have generated a much smaller increase in prices.

This sudden increase in the global demand for goods also generated a sharp increase in the global demand for energy. Goods are more energy intensive to produce than services – think again about the comparison between going to the gym and buying a bicycle. This met a commodity market that was undersupplied for a variety of reasons, including years of underinvestment in fossil fuels, not enough renewable energy capacity and a geopolitical risk premium. The Russian invasion of Ukraine has added to this supply shortage and risk premia. Because energy commodities are a critical input for metals and food, the energy price surge had severe spillovers in the rest of the commodity complex. As a result, global commodity prices have skyrocketed. The Bloomberg commodity index, a broad basket of commodities including energy, metals and food, has appreciated over 100% since the COVID-19 trough in March 2020.

Figure 1

**Core CPI versus core CPI including new and used cars**

% year-on-year

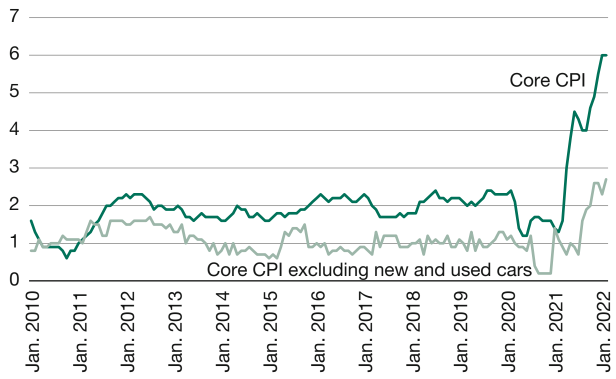

Source: Bloomberg and author's calculations.

These scarcity effects in goods and commodities are, by definition, transitory shocks, though likely very persistent. Figure 2 shows the outlier nature of these price shocks. While US core goods inflation has historically averaged about zero, it is running at well over 10% year on year. Barring new shocks, it stands to reason that goods prices will at least stabilize, and thus their inflation rate will decline to zero. Their inflation rate could also become negative, for example, if the lagged production generated by these scarcity effects eventually results in an excess supply of goods, or if the geopolitical risk priced in commodity markets subsides.

## Erosion of slack: The "great renegotiation"

Of course, the enabling factor for this large array of price shocks is strong demand growth – these scarcity effects would not have happened had demand growth been weak. Demand has been strong due to the expansionary policy mix implemented across all countries, including the very large US fiscal stimulus adopted in early 2021. The differences in fiscal stimulus across countries, and the

3 To wit: US durable goods consumption is 20pp above the pre-COVID-19 level, while services consumption has just recovered the pre-COVID-19 level. This divergence is less acute in the EU, which explains the milder increase in core inflation, as scarcity effects in goods have been much less pronounced (see Lane, 2022).

4 The supply of semiconductors had been disrupted by the US export restrictions introduced by the Trump administration, see Bown (2021).

Figure 2
**US core CPI**

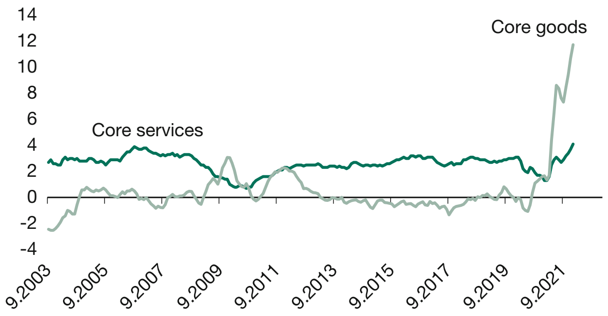

Source: Bloomberg and author's calculations.

Figure 3
**Bloomberg consensus for 2021/Q4**

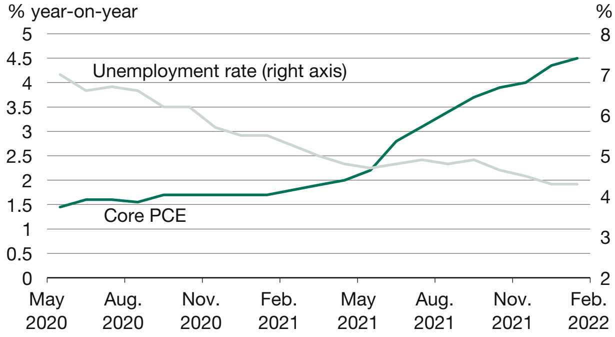

Source: Bloomberg.

differential timing of the reopening of the economy, are factors explaining why the inflation surge started in the US, and only later appeared in the UK and the EU.[5]

But how much inflation did the erosion of slack generate? Was it enough to explain the large upside surprise in core inflation? Slack, defined as the difference between the unemployment rate and the non-accelerating inflation rate of unemployment (NAIRU), is an unobserved variable, but its near term evolution can be proxied by the change in the unemployment rate. Figure 3 shows the evolution of the consensus forecast for the US unemployment rate and for core personal consumption expenditure (PCE) year-on-year in 2021Q4. It shows that as of 2021Q1, the unemployment rate was forecast to end the year at 5.5%, and core PCE was forecast to end at 1.7%. Alas, the unemployment rate fell to 4.2% in 2021Q4 while core PCE rose to 4.5%. In other words, the surprise in unemployment was 1.3 percentage points, while the surprise in core PCE was 2.8 percentage points. Under the Phillips curve specification that prevailed prior to the COVID-19 crisis, which estimated a coefficient of about 0.1 for the unemployment gap (see Reifschneider and Wilcox, 2022), this unexpected decline in the unemployment rate would have generated just about 0.1-0.2 percentage points of additional core inflation.

It is possible that the NAIRU has increased, and that this decline in unemployment represents a bigger erosion of slack. While employment has recovered to pre-COVID-19 levels, the labor force participation rate is still below, likely reflecting a combination of retirements and COVID-19 related hesitancy. Crump et al (2022) estimate the evolution of the NAIRU during the COVID-19 period and conclude that it may have increased to about 5.9% – about 1.5 percentage points higher than pre-COVID-19. That would imply that the surprise in the unemployment gap would be double, about 3 percentage points. Still, with the coefficient of the pre-COVID-19 period, this would have generated additional inflation of only about 0.3 percentage points, a rounding error of the 2.8 percentage point surprise.

It is possible that the Phillips curve has become steeper – that the coefficient of the unemployment gap has increased, thus generating more inflation for a given erosion of slack. But it does not appear so initially. Figure 4 shows the relationship between the unemployment gap (the difference between the unemployment rate and the Congressional Budget Office's estimate of the NAIRU) and core services inflation (the more sensitive part of inflation, a priori, to slack), for the last three expansions (defined as the periods when the unemployment rate was declining). It shows that

Figure 4
**The relationship between core services inflation and slack**

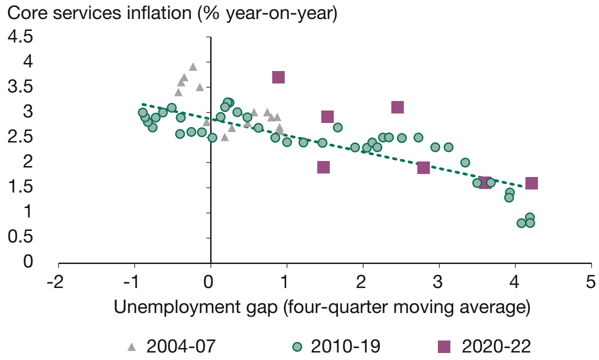

Source: Bloomberg and author's calculations.

5 See Gros (2021) for a discussion of the different fiscal stimulus across countries.

the current episode is for now roughly consistent with the past experience – though the number of observations is small and thus caution is warranted. However, for a steeper Phillips curve to explain the inflation surge, the coefficient would have had to increase tenfold.

The relationship between slack and inflation is driven by two factors: the relationship between slack and wages (the original Phillips curve was a relationship between unemployment and wages) and the relationship between wages and prices, which is driven by markups and pricing power. Thus, this apparent stability of the Phillips curve could be masking moves in these two relationships.

In fact, the rapid increase in wage growth (the Atlanta Fed tracker shows year-on-year wage growth of 5.8% in February 2022) could be signaling an incipient steepening of the Phillips curve. A main driver of this strong wage growth could be the policies implemented in the US to deal with the COVID-19-related lockdowns – rather than furloughing workers and keeping the employer-employee relationships, as most other developed countries did, the US opted for income support via cash checks and enhanced unemployment benefits. This led to a large and sudden increase in unemployment, followed by a large and sudden recovery in employment. This led to the "great renegotiation", with record high amounts of workers searching for new jobs upon reopening. In an environment of strong economic activity, this large-scale renegotiation of contracts has given workers bargaining power to demand higher wages – something that is apparent in the acceleration of wage growth for job switchers, which reached 6.6% in February 2022. This great renegotiation has happened mostly at the lower end of the pay distribution. The Atlanta Fed data shows that while wage growth for the first quartile has accelerated to the highest levels since 2001, wage growth for the second, third and fourth quartiles is broadly similar to the last decade. This acceleration of wage growth for low-wage jobs may have been exacerbated by the restrictions to immigration, as immigrants have traditionally been more willing to fill jobs at the lower end of the pay scale. To a large extent, the observed dynamics of wage growth are similar to a sudden increase in the "market minimum wage" driven by the great renegotiation. As such, it could just be a one-off adjustment in wage levels – though, in the context of a rapid increase in commodity prices, there is a material risk that workers will also want to be compensated for their loss in real incomes, demanding further wage increases and thus triggering a wage-price spiral (see Blanchard, 2022). The lack of unionization in the US labor market reduces this risk, but only time will tell.

At the same time, it is possible that this faster wage growth at the lower end of the pay scale is driven by a fundamental transformation of jobs that is not inflationary. After all, the key variable affecting future inflation is not wage growth, but wage growth relative to productivity. The COVID-19 pandemic has led to a potentially permanent increase in online commerce, as consumers have become used to ordering online and habit formation is typically persistent. This implies an increase in the market share of commercial distribution of Amazon and similar firms, with an important implication: Amazon applies the principles of manufacturing to commercial distribution and can therefore generate higher productivity growth than the in-person retail sector.[6] Thus, a growing share of the services sector is becoming like the manufacturing sector, and thus susceptible to productivity gains. Amazon can afford to pay higher wages that match this higher productivity growth, de facto putting a floor in low skilled wages and generating competitive pressures for the rest of the low wage sector, which must match these higher wages to retain workers and, along the way, increase their productivity if they want to survive. The increasingly monopsonistic nature of the US labor market gives large firms an advantage that exacerbates these effects.

The final element is the markup of prices over marginal costs. Empirically, markups have behaved in a countercyclical manner: During periods of high unemployment, firms benefit from lower wage growth and build large markups, and as the unemployment rate declines and wage growth picks up, firms absorb the increase in labor costs by reducing markups, allowing them to keep prices stable and retain market share. But this countercyclical nature of markups is an empirical fact, not a fundamental property of the economy, and may have been the result of a globalization-driven multidecade trend that limited wage gains and firms pricing power. In an environment where all labor inputs are increasing and all companies are raising prices, it is possible that markups no longer behave in a countercyclical manner, thus increasing the probability of a wage-price spiral.

Overall, the contribution of slack to the inflation surge has likely been very small and, while wage growth has accelerated and pricing power seems widespread, it is too early to tell if it is a one-off or will lead to a permanent increase in inflation.

### Inflation expectations: Increasing from too low levels, now compatible with the inflation objective

The third element of the Phillips curve is inflation expectations. Short-term measures of inflation expectations have increased, mostly the reaction to the increase in food and

6 See the discussion in Ubide (2022).

Figure 5

**US 5-year, 5-year inflation expectations**

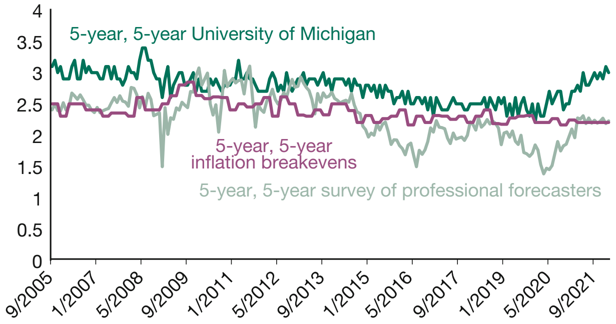

Source: Bloomberg and author's calculations.

Figure 6

**Estimates of the adaptive component of US inflation expectations**

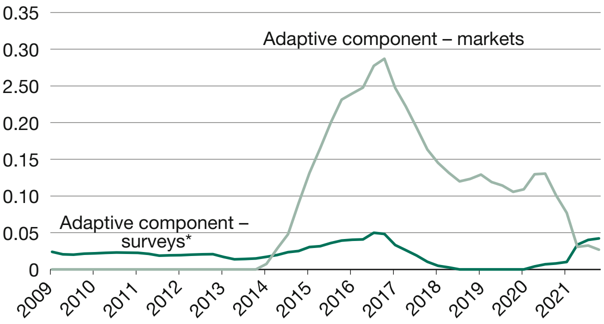

Notes: *Survey of professional forecasters and University of Michigan: Inflation Expectation.

Source: Bloomberg and author's calculations.

energy, the most salient prices. But short-term inflation expectations have not, in the past, been a good predictor of inflation: Reifschneider and Wilcox (2022) show that one-year-ahead inflation expectations have been negatively correlated with inflation one year out. Longer-term measures of inflation expectation are more relevant for future inflation and have increased from the excessively low levels that were prevailing pre-COVID-19, but have stabilized at levels compatible with the 2% inflation objective. For example, Figure 5 shows the evolution of US 5-year, 5-year inflation expectations derived from three different approaches: survey of professional forecasters, consumer surveys and financial markets. These three measures are at levels similar to or below the levels prevailing in the 2004-2007 period, when there was widespread consensus that price stability had been achieved.

However, the breath of the surge in prices could cast doubt on the underlying stability of measured inflation expectations. If inflation expectations were to become more adaptive (more backward looking, reacting more to the recent inflation prints), then the anchoring of inflation expectations would be at risk. Figure 6 shows the estimates of the adaptive component of US inflation expectations, from a model that decomposes the evolution of 5-year, 5-year inflation expectations as a function of forward-looking and adaptive components.[7] It shows that while there was an increase in the adaptive component during the 2014-19 low inflation period, reflecting deflationary fears, there is little evidence now that long-term inflation expectations are becoming adaptive, reflecting inflationary fears. It is comforting that, despite the 40-year high in measured inflation and the widespread and salient increases in prices, long-term inflation expectations remain well anchored – if not now, when would inflation expectations de-anchor, one could ask. But vigilance is certainly warranted as the tails of the distribution of market-based inflation expectations are widening (see Hilsher et al., 2022).

## Implications for monetary policy: A different policy strategy

Overall, the conclusion is that there is, for now, little information in the current acceleration of inflation that casts doubt on the future stability of inflation. But inflation is, after all, an economic policy phenomenon, and thus this conclusion is critically dependent on the continuation of a policy mix, both monetary and fiscal, that ensures that inflation expectations remain well anchored so that, if and when the inflation shocks fade, inflation can return to target. The concept of the policy mix is critical: Fiscal policy is the right instrument to address the hit on incomes from the price surge, allowing monetary policy to adjust accordingly.

What should monetary policy do? With inflation expectation at target and the labor market very close to approaching maximum employment, monetary policy must now adjust and increase interest rates towards neutral levels. For as long as inflation expectations remain well anchored, this can be achieved with a series of rate increases and a clear commitment to do whatever it takes to ensure price stability. Forward guidance is a symmetric tool that should also be used during tightening cycles. In fact, by the time the Fed started to raise rates in March 2022, 30-year mortgage rates had already increased by 150 basis points since mid-2021, tightening financial conditions. Forward guidance also operates in the risk space in a cyclically adjusted manner (see Ubide, 2017). During times of low inflation, forward guidance contributes to re-

7 The model decomposes inflation expectations as a function of lagged inflation and the central bank target, with time varying betas that are constrained to sum to one. It is estimated via a five-year rolling regression.

laxing the effective lower bound (ELB) constraint by conveying certainty about the future rate path and promoting risk taking. During periods of high inflation, forward guidance can contribute to tightening financial conditions by limiting the information about the future pace of rate increases and limiting risk taking.

The current tightening cycle will be, by design, different from past cycles. In the past, monetary policy was preemptive – it started to tighten policy while the unemployment rate was still above the NAIRU. For example, the Fed started raising rates in 2015 with the unemployment rate at 5.1%, about 1.5 percentage points higher than the 3.5% level achieved in 2019, and with core PCE at 1.1%, well below the 2% objective – so that the tightening cycle would be gradual. However, in doing so, monetary policy was, de facto, treating the inflation objective as a ceiling, not a symmetric target, and the outcome was suboptimal, with inflation and inflation expectations staying below target. The innovation of this cycle, in part by design but also in part due to the surprisingly strong recovery, is that monetary policy has sought to overcome the ELB constraint by no longer being preemptive, waiting to start the tightening cycle until the unemployment rate had reached the NAIRU and inflation was at target. Therefore, the tightening process starts later in the cycle than before. But this also means that it can and will likely be less gradual.

But can monetary policy deliver a faster tightening process and yet also achieve a smooth landing? Some Fed tightening cycles have ended in recession, but others have not. History may not be a good guide: There are few observations (just seven tightening cycles since the 1980s) and the initial conditions, productivity trends and shocks of each cycle have been different. For example, during the 1980-2000 period, the Fed engaged in a strategy of opportunistic disinflation, with the objective of starting each expansion with a lower level of inflation than that prevailing at the peak of the previous cycle. This means that each tightening cycle aimed at lowering the level of trend inflation, not just stabilising inflation at target. And this required, most of the time, a recession to shift the inflation dynamics lower. The 2004-07 episode ended with a financial crisis, and the 2015-18 episode ended with the COVID-19 recession.

This time is also different because the Fed only wants to stabilize inflation and inflation expectations at target, not reduce the trend level of inflation. If inflation expectations remain anchored and supply shocks fade, the Fed can achieve this by raising rates to a bit above neutral levels and stabilize the unemployment rate at the NAIRU. Once there, the Fed must decide whether it wants to keep inflation at 2% or, considering that inflation typically declines during recessions, stabilize core inflation in the 2%-2.5% range so that it averages 2% over the business cycle. This strategy of opportunistic reflation (as suggested in Ubide, 2017) would help overcome the ELB constraint and boost future growth.

In the end, central banks must accept that a symmetric inflation objective requires taking some upside risk with inflation. Let us not forget that many central banks, especially the ECB, have struggled since 2007 to lift inflation to target. There is a clear trade-off: the less upside inflation risks are accepted, the more the inflation objective is a ceiling, and not a midpoint, and the more future growth is foregone.

## Conclusion

The surge in inflation has been due mostly to large and widespread price level shocks, in a context of strong demand growth and anchored inflation expectations. As such, there is little information in the current inflation surge that suggests this increase in inflation will be permanent. Conditional upon the right fiscal policy response to cushion the hit on incomes – this is more critical in the eurozone, where the shock from the Russian invasion of Ukraine is large and inflation expectations are still fragile after a decade of very low inflation, and it is therefore too early to declare mission accomplished on the re-anchoring of inflation expectations at the 2% target – a well-managed adjustment of monetary policy should be able to stabilize inflation at the target. The current inflation environment is extraordinary and will require nimble and symmetric decision-making. There is no room for complacency, but no need to panic either.
